# The acceptability of integrated healthcare services for HIV and non-communicable diseases: experiences from patients and healthcare workers in Tanzania

**DOI:** 10.1186/s12913-022-08065-4

**Published:** 2022-05-16

**Authors:** Elizabeth H. Shayo, Sokoine Kivuyo, Janet Seeley, Dominic Bukenya, Peter Karoli, Sayoki Godfrey Mfinanga, Shabbar Jaffar, Marie-Claire Van Hout

**Affiliations:** 1 National Institute for Medical Research, Dar es Salaam, Tanzania; 2 MRC/UVRI & LSHTM Uganda Research Unit, Entebbe, Uganda; 3 Department of Global Health and Development, London School of Hygiene and Tropical Medicine, London, UK; 4 Liverpool School of Tropical Medicine, Liverpool, UK; 5 Liverpool John Moores University, Merseyside, UK

**Keywords:** Acceptability, HIV, Non-communicable diseases, NCD; integrated care, Patients, Providers, Tanzania

## Abstract

**Background:**

In sub-Saharan Africa, the prevalence of non-communicable diseases (NCDs) has risen sharply amidst a high burden of communicable diseases. An integrated approach to HIV and NCD care offers the potential of strengthening disease control programmes. We used qualitative methods to explore patients’ and care-providers’ experiences and perspectives on the acceptability of integrated care for HIV-infection, diabetes mellitus (DM), and hypertension (HT) in Tanzania.

**Methods:**

A qualitative study was conducted in selected health facilities in Dar es Salaam and Coastal regions, which had started to provide integrated care and management for HIV, DM, and HT using a single research clinic for patients with one or more of these conditions. In-depth interviews were held with patients and healthcare providers at three time points: At enrolment (prior to the patient receiving integrated care, at the mid-line and at the study end). A minimum of 16 patients and 12 healthcare providers were sampled for each time point. Observation was also carried out in the respective clinics during pre- and mid-line phases. The Theoretical Framework of Acceptability (TFA) underpinned the structure and interpretation of the combined qualitative and observational data sets.

**Results:**

Patients and healthcare providers revealed a positive attitude towards the integrated care delivery model at the mid-line and at study end-time points. High acceptability was related to increased exposure to service integration in terms of satisfaction with the clinic setup, seating arrangements and the provision of medical care services. Satisfaction also centred on the patients’ freedom to move from one service point to another, and to discuss the services and their own health status amongst themselves. Adherence to medication and scheduling of clinic appointments appeared central to the patient-provider relationship as an aspect in the provision of quality services. Multi-condition health education, patient time and cost-saving, and detection of undiagnosed disease conditions emerged as benefits. On the other hand, a few challenges included long waiting times and limited privacy in lower and periphery health facilities due to infrastructural limitations.

**Conclusion:**

The study reveals a continued high level of acceptability of the integrated care model among study participants in Tanzania. This calls for evaluation in a larger and a comparative study. Nevertheless, much more concerted efforts are necessary to address structural challenges and maximise privacy and confidentiality.

## Background

In Sub-Saharan Africa, the population growth and rising life expectancy coupled with rapidly changing lifestyles have resulted in increased levels of non-communicable diseases (NCDs) in contexts where there is already a high burden of HIV infection [1]. Among adults in the region, the prevalence of diabetes mellitus (DM) is 4–5% whereas hypertension (HT) is above 25% and the HIV prevalence is 5–20% [2–4]. Some studies have reported increased HT and DM risks among people living with HIV (PLHIV) in Africa. For example, out of 912 PLHIV in Malawi, 26.6% had HT and/or DM [5]. In Tanzania, where our study was conducted, the overall estimated prevalence of DM is 9.1%, HT 26% [6, 7] whereas HIV is 5% [8], contributing to chronic disease levels demanding long-term care. Furthermore, 306 long-term ART clients recruited in Dar es Salaam had 25.2 and 17% HT and DM, respectively [9]. These overlapping conditions warrant a co-ordinated health response [10].

Many people with DM and HT face a poor quality of care for managing their co-morbidities in sub-Saharan Africa, which affects their treatment continuum and related health outcomes [11]. In contrast, PLHIV in the region are in regular care and receive free healthcare services unlike those with DM and HT [3, 4, 12]. Services for HIV and NCDs are provided in standalone clinics with separate waiting areas, consultation rooms, pharmacies, drug procurement chains, and financing. This approach leads to service duplication, with standalone clinics ill-equipped to care for multi-co-morbid patients. In Kenya, PLHIV with co-morbidity face service accessibility and affordability as well as access of competent medical care challenges [13].

Integration of services for chronic conditions into a single clinic model offers a cost-effective model for treating and caring for patients with co-morbidities [14, 15]. The model reportedly account for enhanced efficiency in different low- and middle-income countries [10, 16, 17]. In Africa, patients in Malawi in need of cervical cancer and HIV treatment have benefited from reduced duplication of care and unnecessary travel as a result [18]. Studies on integrating health services in Ethiopia, Swaziland and South Africa revealed positive outcomes, such as high patient retention rates, improved health conditions and cost-effectiveness [19–21]. A study in Kenya on HIV and NCD care reported high perceived acceptability in a medication adherence club due to patient cost-saving and reduced waiting times as all medicines were collected at once in the club [13].

In Tanzania, where we piloted an integrated model of HIV and NCD care to-date little is known about its acceptability among patients and healthcare providers. We adopted the following definition of acceptability by Sekhon and colleagues [22] :4; ‘*a multi-faceted construct that reflects the extent to which people delivering or receiving a health care intervention consider it to be appropriate, based on anticipated or experienced cognitive and emotional responses to the intervention’* and the definition by Proctor [23] who employs the term `acceptability’ in instances where a ‘*treatment, service, practice, or innovation is agreeable, palatable, or satisfactory’*. We utilised a qualitative approach to explore and observe patients and health care providers experiences and perspectives regarding aspects of the acceptability of integrated care for HIV, DM, and HT in Tanzania.

## Methods

In this study we adhered to the consolidated criteria for reporting qualitative research (COREQ): a 32-item checklist for interviews and focus groups [24].

### Study design

**Fig. 1:**
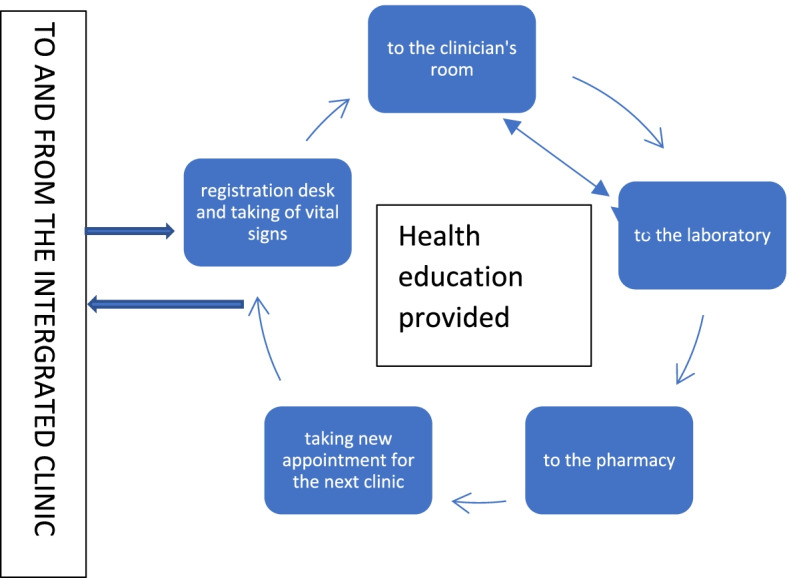
Structure and pathway in an integrated clinic

This qualitative study was nested in a bigger feasibility programme of work conducted in five selected facilities in Dar es Salaam and Coastal (Pwani) regions in Tanzania. The programme entitled *“Models of Chronic Care in Africa”* (MOCCA) aimed to develop and evaluate a model of integrated HIV and NCD care for providing all requisite services in a single location as a *‘one-stop centre’* (see Fig. 1). They hold one patient’s record managed by the same clinicians, nurses, counsellors, and other staff; access one pharmacy with integrated dispensing; and their laboratory samples managed and tested in the same laboratory service (where possible).

Healthcare providers were trained on providing quality and standardised care for all conditions in an integrated manner. The MOCCA project also provided a buffer supply of drugs to the ‘one stop’ facilities in an event of drug shortages [25].

Interviews were conducted with patients and healthcare providers during three time points of the MOCCA project: pre delivery of the one stop service (Month One); shortly after integrated care was provided (Months Three to Five), and at the end of the study (10 months). Hereafter named as ‘pre-phase, mid-line and end-line’. The three data collection points helped to explore whether the acceptability of the experience in the ‘one-stop’ clinic among patients and healthcare providers changed with increasing exposure and continuity of care within an integrated setting. Qualitative data collection was supported by clinic observations at the pre- and mid-line phases (clinic location, seating arrangements for patients, service flow to different service points). The pre-phase observation shaped the integration model whereas the mid-line (soon after the integration) observation helped to improve areas that were insufficiently established as planned.

### Study setting

The study purposively selected health facilities in the Dar es Salaam and Coastal regions, representing different levels, ownership, and rural-urban characteristics. We selected four out of five health facilities (Amana, Hindu Mandal as urban hospitals, Mkuranga as a periphery hospital, and Bunju as a lower-level facility).

### Sample size and recruitment of study participants

Study participants included patients seeking care and healthcare providers. The first author conducted recruitment, with assistance from project co-ordinators and clinic in-charges in their respective health facilities. From each facility, a minimum of five patients with either HIV, HT, or DM as well as those with different co-morbid conditions (HIV/DM, HIV/HT, HIV/DM/HT, DM/HT), and who benefited from integrated care were recruited for interviews (pre-phase, mid-line, and end-line) using convenience sampling. Moreover, a minimum of three healthcare providers were purposively recruited from each health facility (nurses, clinicians, and in-charges of the clinics); at the pre-phase, these cadres were recruited from their independent standalone clinic (NCD and HIV) whereas at the mid-line and end-line, they were recruited in the integrated clinics. More healthcare providers were interviewed at pre-phase than mid-line and end-line levels, and recruitment continued until data saturation was reached.

### Data collection techniques

#### In-depth interviews (IDIs) with health care providers

In-depth interviews were held with the healthcare providers managing clinics (NCD, Care and Treatment Centre-CTC) and Out-Patients Departments [OPDs] at the pre-phase), and at the integrated *‘one-stop’* clinic at the mid- and end-line phases. Insights gained during the pre-phase guided the structuring of the integrated *‘one-stop’* clinic. At the mid- and end-line, interviews captured the realities and the lived experiences in delivering integrated care with a focus on the challenges, quality of care and benefits for both healthcare providers and their patients relative to previous stand-alone condition specific clinics.

#### In-depth interviews with patients

In-depth interviews with patients occurred after they had received clinical management of their conditions and provided post-informed consent. Interviews explored the perspectives of patients on their condition(s), views on integration in the pre-phase, and their experiences of clinic attendance, quality of services received, benefits and challenges.

A trained team of social scientists conducted the interviews which occurred in private spaces around the stand-alone clinics in the pre-phase and ‘*one-stop’* clinics during the integration. They were audio-recorded after obtaining consent from the study participants, each lasting between 45 and 60 minutes (See Table 1).

**Table 1:** Summary of qualitative data collection

Data collection technique	Disease conditions and number of interviews				
	PLHIV	DM	HT	Combination	Total
**Baseline: Pre-phase**					
In-depth interviews with patients	3	3	3	13	22
In-depth interviews with health care providers	5 in-charges, 5 clinicians, 6 nurses working at the CTC, NCD or OPD				16
Observations	8 from independent clinics (NCD and CTC)				
**Soon after integration—midline**					
In-depth interviews with patients	5	5	5	5	20
In-depth interviews with care providers	4 in-charges of integrated clinic, 4 clinicians and 4 nurses both working in the integrated clinic				12
Observations	4 from integrated clinics				
**Post integration—end-line**					
In-depth interviews with patients	5	1	1	8	15
In-depth interviews with care providers	Composed of in-charges of integrated clinic, clinicians, and nurses				12

#### Observations

Observations were made in all the clinics (CTC and NCD) for the hospital level facilities and at OPD for the dispensaries during the pre-phase and in mid-line (soon after the integration had started). The observations focused on the environment of the clinic location and the structure, the freedom in participating in discussions and in moving from one point to another (registration, consultation room, laboratory, and drug dispensing room) and how health education was provided etc. The first author did the observations.

#### Data management and analysis

The theoretical framework of acceptability (TFA) [22] underpinned the structure and interpretation of the qualitative and observational data. The seven constructs of the TFA are as illustrated in Table 2.

**Table 2:** Theoretical framework of acceptability constructs and codes

Construct	Meaning	Codes from the findings
**Affective attitude**	How an individual feels about the intervention	Satisfaction with integrated model and its pathways
**Ethicality**	The extent to which the intervention has a good fit with an individual value system.	Client provider relationship, privacy, and confidentiality
**Intervention coherence**	The extent to which the participant understands the intervention and how it works.	Understanding of the model design and services delivered
**Perceived effectiveness**	The extent to which the intervention is perceived as likely to achieve its purpose.	Availability of quality of services; increased awareness and improved health status
**Self-efficacy**	Participant ‘confidence’ that they can perform the behaviour required to participate in the intervention	Comfort with sitting arrangement, freedom of movement; making discussion, fixing clinic appointment and medicine adherence
**Burden**	Amount of effort that is required to participate in the intervention	waiting time; cost related to services and time saving; and medicine availability
**Opportunity cost**	The extent to which benefits, profits or values must be given up engaging in the intervention.	Transport costs, distance, access to medicines; changing of clinic schedule; managing multiple information system

Ref: Sekhon and colleagues [22]:8

Verbatim transcription was done by social science research team members who later translated from Kiswahili into English. Using the thematic content analysis, guided by the TFA, the first author carefully read all the transcripts and listened to all the recordings to become immersed in the data, and confirm the correctness of the transcription. Comparison of the notes on the patterns observed in the data and emerging themes was made by the whole team and developed a common coding frame. Two social scientists, who collected the data were involved in coding under the supervision of the first author. The observational data was combined with qualitative and analysed thematically. An excel sheet was created for all information in the transcripts relevant to the codes developed, copied, and pasted, hence facilitating the observation of patterns, similarities, and differences of responses between different levels and type of informants. Categories were clustered based on TFA constructs that structured the presentations of findings. The team approach analysis and interpretation was supported by the third, fourth and last authors. Triangulation of data across methods and sources was subsequently undertaken. i.e., the observational data was combined with in-depth interviews and analysed thematically. This approach enhanced the trustworthiness and validity of the information collected.

## Results

The findings are presented under seven constructs of the TFA: Affective attitude, Self-efficacy, Burden, Perceived effectiveness, Opportunity costs, Ethicality, and Intervention coherence. We present the analysis within each construct as flowing sequentially from pre to mid and to end-line, divided into distinct sub themes.

### Affective attitude

#### Satisfaction with the integrated model

At the pre-phase, most of the patients and health care-providers exhibited and described a positive attitude to the integrated clinic model., i.e., patients showed their willingness to attend and receive services from the integrated clinic and healthcare providers were ready and happy to deliver services, reflecting anticipated affective attitude. The following are baseline testimonies: “*… I would prefer PLHIV and other disease conditions to be managed by a single doctor. I think it will be much better …*” (PLHIV with HT, female, hospital-urban, pre-phase). Healthcare providers supported the integration concept and called for existing structures to manage PLHIV be suitable to implement the integrated model.

During the integration, positive feelings towards integration were revealed by patients and care providers which increased as they continued being exposed to the integrated care model. Most of the patients at mid-line and end-line phases reported satisfaction with the pathway as all services were provided in a single clinic setting (as shown in Fig. 1), restricting unnecessary movement. They declared easiness in following the procedures. Satisfaction was attributable to service provision operated on the principle of ‘first come first served’. At the end-line, patients were informed that the project had concluded and had to indicate whether they would like to remain in integrated care. A substantial consensus emerged as most patients were willing to continue with their care in an integrated clinic:

*“Integration is good. At first, I disagreed [with the concept] but now I see that it is good; I am ready to continue with the clinic and encourage other patients to attend such an integrated clinic*” (Patient with HT/DM, female, periphery hospital, end-line).

Participants with multiple conditions reported greater satisfaction. Examples included a female patient with HIV and HT from the hospital level indicating that she would drop out of the services should she return to the standalone clinics. All patients indicated that they would advise their friends and family members to join the *‘one stop’* clinic:

### Self-efficacy

Self-efficacy ranged from comfort with the sitting arrangements, movement from one point of care to another, freedom of discussion, fixing clinic appointments and medicine adherence. During the baseline, when patients were receiving services in their respective standalone clinics, some of them with NCD conditions reported difficulties in sitting together with PLHIV. They worried about the possibility of being identified as Patients with HIV and contracting other conditions related to HIV infection such as tuberculosis:

*“I will feel bad sitting together with affected ones [HIV], how will people understand me, I am not one of them, because everyone knows this clinic is for HT, DM, and HIV, other people will wonder ‘What is wrong with this woman?’* (Patient with HT and DM, female, hospital level, pre-phase)**.**

The same concern emerged among healthcare providers who reported that they were unsure about whether PLHIV would be willing to mix with patients with other disease conditions as they were managed privately to ensure confidentiality.

The observation at the pre-phase revealed the service points such as laboratory and medicine dispensing were located in a different area, adding more movement to patients. During integration, the CTC clinic served as a point for integration and the same waiting area was used for all patients regardless of disease conditions, and an acceptable level of comfort with the sitting arrangement was mostly reported by patients from the hospital level in the urban area for both mid-line and in end-line data collection points. Patients could sit anywhere in the integrated clinic and were comfortable to move from one point to another while following the instructions given. This was confirmed by health care providers who reported that they were stringent with queue observance.

When waiting for the services, most of the patients described a sense of freedom in discussing their health status, how they felt, side-effects of their medication, and even reported encouraging others not to withdraw from the integrated ‘*one stop’* clinic. Healthcare providers said that free discussion among patients helped to improve health literacy across all three conditions, and was deemed to support positive health behaviours and medicine adherence:

*“We provide health education to the patients on all three disease conditions in a single clinic which has integrated all three services in one place rather than having two separate clinics. We provide education to eliminate stigma, and this helps them to come together”* (Healthcare Provider, hospital level, mid-line).

At the mid-line when the integrated care services were recently initiated, healthcare providers declared difficulties in providing health education covering the three disease conditions in the same session as they were not used to doing so in standalone clinics. The observations revealed the same where patients with HT and DM and HIV received health education separately in their respective standalone clinics before engaging in the integrated clinic for other services. However, as healthcare providers gained more understanding on how to balance health messages for the three disease conditions these perceived difficulties were substantially reduced. Despite the positive reflections, few patients with HT and DM in the lower and periphery facilities reported dissatisfaction with the integrated clinic set-up like in the baseline. They persistently mentioned being uncomfortable with being mixed with *PLHIV* in the waiting area during both the mid-line and end-line.

#### Fixing clinic appointments/refill and adherence to the medicine administration

At the baseline, patients reported dropping out of the services, especially in the NCD clinics because of a poor therapeutic relationship between patient and healthcare provider, negligence in medicine uses due to alcohol consumption and irregularity in the medicine availability, the information that was also supported by healthcare providers. The recurrence of symptoms instigated a return to care.

At the mid-line, most patients declared that they had adhered to their clinic appointments and medicine administration according to the advice they were given, health education provided ought to serve as a motivating factor;

*“I adhere to the treatment very well. I do take the medication on time as prescribed and follow the doctor’s instructions. I adhere to my doctor’s advice very well that is why I am doing very well”* (Patient with DM and HT, male, lower-level facility, mid-line)

Despite the high level of adherence, care providers reported to have encountered challenges for few patients with NCDs in adhering to medicine regimes advice. At the end-line, however, all the patients interviewed across all disease conditions confirmed adherence to treatment regimens and attendance at regular clinic appointments. Healthcare providers were also affirmative: “*Yes, clients show good adherence to treatment, and they are doing better; they stay in the treatment”* (Healthcare Provider, hospital-urban, end-line).

### Burden

At the pre-phase, Patients with NCDs complained about waiting times because of the need to seek services at different points within the health facilities, an occurrence which was also observed by researchers. Also, patients presumed minimal effort was required to participate in the intervention, the assumption that was confirmed in midline and end-line phase. For example, patients with co-morbidity conditions either HIV/HT/DM or HT/DM supported the idea of integration as it was perceived to save time and costs of visiting multiple clinics. A positive reflection was also provided by healthcare providers at all levels of the health facilities whether dispensary or hospital, rural or urban:

*“I am 100% satisfied because integration will enable PLHIV with also DM and HT to receive the services under the same roof to reduce disturbances of moving from one clinic to another.*” (Health Care Provider, hospital level, pre-phase).

As it was predicted at the pre-phase, availability of all services in a single location as ‘one stop centre’, cost and timesaving were identified by patients and care providers as the main factors for increased acceptability of the integrated model because of the reduced burden.

The model was viewed by all the study participants as helping to reduce inconvenience for patients with multiple disease conditions who used to attend different stand-alone clinics at different appointment dates. The observation also revealed the ease with which patients could access care at different points as they were arranged in the same building, hence reducing unnecessary movement, as was noted in the pre-phase. The findings from the patients were confirmed by care providers.

Reduced financial burden and time saved enabled patients to engage in economic activities as it was confirmed by patients and care providers at the midline and end-line phase. Most patients with NCDs declared medicine availability at the mid-line and end-line to differ from the pre-phase.

Despite the positive reflections, most patients had concerns over waiting time, mostly during the mid-line phase because of the shortage of human resources and some services were not yet in a single location which was also observed by researchers. The registration and health education were still taking place in the independent clinic. Such complaints diminished as they entered the end-line phase.

### Opportunity costs

At the pre-phase, patients with HT or DM described their experiences of attending a single treatment clinic and how they could not receive appropriate care:

“*The doctor has no time to listen to me at all especially the one responsible for HT. She is busy with her mobile phones. Just imagine, we come very early in the morning without eating anything. I have made some calculations that I pay 10,000Tshs [$5] to the provider who has no time with me!”* (PL HIV/HT, female, hospital level, pre-phase)

In the in mid-line and end-line phase, study participants also reported transport costs and lack of specific medicines for HT and DM as challenges, but patients continued attending their clinic sessions and would later purchase medicines from other places.

Some patients reportedly changed their clinic schedule from 3 months to a monthly basis to facilitate close monitoring in the integrated clinic. However, this was difficult for some patients who failed to balance work and clinic attendance:

“*The work environment is sometimes hard for me e.g., the clinic appointment date might overlap with my work-shift which is difficult to change. This may necessitate requesting the change of the clinic day or sometimes when I am from the work I am forced to go direct to the clinic”* (Patient with HIV/DM, male, hospital level, midline phase).

Healthcare providers further said that during provision of the ‘one-stop’ clinic different health information systems existed for HIV and NCDs, hence necessitating improvising the registers to facilitate recording of patients’ information, and later navigate into the hospital records system. This was also observed by researchers.

### Perceived effectiveness

Since the *‘one-stop’* integrated care intervention aimed to improve service delivery provision, perceived effectiveness focused on the quality of diagnostic facilities, trained staff and increased awareness and health status.

#### Availability of services in the integrated clinic

During the baseline, patients with either DM or HT reported insufficient services that they had received in their NCD clinics. As health care providers were introduced about the integration, they advised the MOCCA project to ensure the availability of medicine, trained providers, and diagnostic tools prior to operationalisation of the ‘one-stop’ clinics.

When they entered the integrated *‘one-stop’* clinic most of patients reported an improvement at mid-line, which significantly increased at the end-line phase as also confirmed by healthcare providers:

“*We do provide all the patients with health education every morning, consultation, laboratory investigation and dispensing medicines and advice on medicine adherence. Specifically, for HIV we do dispense anti-retroviral therapy; undertake viral load testing, for HT; we do measure blood pressure, height, weight, dispense anti-hypertensive medicines and for DM; we do measure RBG and dispense metformin and Gemma 2*” (Healthcare Provider, lower-level facility, mid-line).

Majority of patients described how the screening tools for taking vital signs including measuring of glucose level were available and that clinicians provided an enhanced prescription on the medication as per disease conditions.

At midline, however, few patients with HT and DM reported that the integrated care lacked diagnostic and monitoring tools as well as clinical skills:

*“Screening is not good as it takes a long time and, sometimes, the tools are unavailable, medicines are unavailable … . We do not have specific doctors; any doctor can attend us. We had our own doctors from the places we came”* (Patient with DM, male, periphery hospital, mid-line).

The complaints were confirmed by several health care providers about the scarcity of trained providers to deliver integrated care and the occasional unavailability of random blood glucose (RBG) strips. However, the situation improved as they entered the end-line phase.

#### Improved awareness and health status

Provision of health education was reported to have raised awareness among patients to check frequently for other conditions. They were provided with good advice on how to improve their health status, hence preventing further risks and complications. All patients generally reported an improved health status overall and were happy that other disease conditions could be detected earlier. Patients whose blood pressure was uncontrolled were reported to have benefited. Healthcare providers also confirmed this:

*“There have been significant benefits; patients were previously not known to be hypertensive and diabetic but now they have been identified and have started the medication. Moreover, they adhere to their medication and lifestyle modification … [As a result] PLHIV have achieved viral load suppression and are currently stable* (Healthcare Provider, lower-level facility, mid-line).

### Ethicality

#### Client-provider relationship

Before the integration of care into the ‘*one stop’* clinic, some patients at the NCD clinics reported being less satisfied as health workers were perceived to pay little attention to them when seeking care. However, with increasing duration in the integrated clinic, these complaints reduced over time. Most patients reported a good relationship with the healthcare providers, which was observed to significantly improve as they entered the end-line phase.

Patients described the use of good language, provision of advice and care, and receiving health education as some of the positive attributes: “*Honestly the relationship between the patient and the health providers is good because they received me well”* (PLHIV, female, hospital level-urban, end-line). Healthcare providers also confirmed this positive relationship to include the use of friendly language and ensuring that only a reasonable number of patients were admitted in a single clinic visit to avoid congestion and long waiting times. They called for the building of trust for patients and ensuring a friendly and conducive environment:

*“At the individual level, we make sure that patients are warmly welcomed, and that good rapport is established. At the facility level, we ensure a proper sitting plan in the patients’ waiting area. We encourage them and make sure that all the necessary investigations and essential medicines are available”* (Healthcare Provider, periphery hospital, mid-line).

#### Privacy and confidentiality

The findings at the pre-phase revealed the differences between the clinics managing PLHIV (CTC) and those with HT and DM regarding aspects of privacy and confidentiality assurances. The NCD clinics had small waiting areas with poor ventilation, the exact opposite of what was reported by patients regarding the CTC building.

During the integration of care in ‘*one-stop’* clinics, some patients with HT and DM from lower and periphery health facilities perceived integrated services to have infringed upon their privacy. They indicated that the integrated clinic CTC) areas was so open that anyone could see them when entering the clinic and patients reported congestion in the small patients’ waiting area, also confirmed by observational findings. Social stigma was therefore reported by Patients with NCDs as a factor affecting acceptability which was also confirmed by health care providers;

*“It has been difficult to accept these integrated services by some of the patients especially those with either DM or HT. They complain a lot that … that if other people see them, they would be misconstrued to also be HIV-positive cases. Our environment is not friendly; it’s too open there is no privacy...”* (Healthcare Provider, lower-level facility, mid-line).

Some providers noted that there was an additional problem as a single room served all the clinicians, who would sit there and consult with patients which jeopardized privacy and confidentiality.

### Intervention coherence

At the mid-line, most patients indicated increased understanding of the services delivered in the integrated clinic. They mentioned receiving services following the patient pathway from file taking, registration, measuring of vital signs, health education, consultation, laboratory and collecting medicines. Increased understanding of the importance of integration and delivery of quality services emerged to be an added advantage for the increased acceptability of the integrated *‘one-stop’* clinic concept over time.

*“I now have a good understanding of the services provided at this integrated clinic. The service providers are good at providing services, which are also good. I am willing to continue receiving treatment at this clinic”* (PLHIV with HT and DM, female, lower-level facility, mid-line).

All healthcare providers supported the continuation of such integrated health services because of the accruing benefits. Other reasons included a good relationship that had been cultivated between health care-providers and their patients, and cost-saving in terms of transport expenses and the availability of medicine.

## Discussion

The study yields unique insights into the experiences of patients and health care providers receiving and delivering integrated services for HIV and NCD care in Tanzania. We found that both patients and care providers have affirmed the development of a positive attitude to the integrated ‘*one stop’* model over time. Most patients were free to enter the clinic, move from one service point to another and fix clinic appointments. They acknowledged the cost and time saving benefits, as well as increased awareness and detection of other disease conditions that were previously undiagnosed. These findings were supported by healthcare providers. However, at the start of the intervention (mid-line), some dilemmas emerged as some of the services were not yet available in the same location. There were few staff allocated to integrated clinics who performed dual roles. These challenges were quickly fixed by the health facility management in collaboration with the project co-ordinating team. In this study, it was also evident that integrated care provision was inseparable from improved quality of services. Indeed, improved client-provider relationship, improved medicine availability especially for NCDs inevitably enhanced retention.

A recent scoping review in lower and middle income countries has also suggested that stakeholders’ opinions on recommending treatment intervention to a friend or family member helps to determine acceptability and support for the feasibility of an intervention [17]. Other studies have also called for evaluating the acceptability and the negative impacts of an integrated model in specific contexts [26, 27]. Our study has found positive reflections on these aspects, essential for scalability. The willingness also featured in other studies in Uganda and the Democratic Republic of Congo where patients preferred to receive HIV services in TB clinics [28]. In low and middle income countries, integrated care could offer preventive, screening and therapeutic services to patients with NCDs [29], and the HIV clinic setting is an optimal point for integration [30]. The strong acceptability evident in our study is based on their understanding of the integrated model and expected services that were met as intended, qualifying the intervention coherence construct of the TFA.

Applying the TFA requires appreciating the overlap of constructs, as such, interpretation of study findings calls for a multi-facet lens since they fit in more than one construct. For example, issues of privacy, confidentiality, waiting times, medicine availability, cost and time saving, increased knowledge and possibility of detection of other disease conditions.

Even though at the baseline, there was fear about mixing PLHIV and those with NCDs, during integration most patients at midline and end-line reported being comfortable with the sitting arrangement, making discussion and moving from one service point to another. The confidence of attending the integrated care services is confirmed by the extent to which patients scheduled clinic appointments and adhered to medicine regimens. This was attributed by a good client provider relationship as also noted in HIV/TB integration study in the Democratic Republic of Congo [28], and health education on the possible benefits for their health. Improved adherence was also noted in studies in Uganda [31]. The maximum level of drug adherence and scheduling of clinic appointments was difficult to achieve prior to integration where the services were provided vertically in the independent clinics. In other words, the ethicality construct of the TFA explains the high retention observed in the feasibility study in the clinical setting among all participants with any one or more of these conditions (HIV, DM and/or HT) [25]. A similar observation was found in a study conducted in Kenya where NCD patients attending the medication adherence clubs reported adherence of 99% [32]. In our study, only a few patients with NCDs doubted integrated care mainly because of the structure in lower level and periphery facility that maintained limited privacy, a finding also noted in TB/HIV integration in the Democratic Republic of Congo [28].

Overall, the integrated clinic care pathways could be more acceptable if overcrowding were reduced, and privacy observed at the different care pathway points at the lower level and periphery health facilities. Consistent with this finding is a study from Malawi, which reported that barriers of the integrated clinic included limited physical space as patients waited to access the care and unavailability of medical supplies [18, 28, 33].

Our study further show that the integrated clinic was acceptable due to the high-quality service provided to patients. The model enhanced the provision of multi-condition health education spanning HIV and NCDs stimulating positive health behaviour changes and yielding an improvement in their health conditions. Also, clients with comorbidities spent less money on transport because they were not required to make frequent independent medical visits to the health facility, which had a positive effect on day-to-day life of patients because of the reduced burden. The effectiveness of the integrated model in enhancing detection of other conditions and NCD prevention efficiently and cost-effectively has been reported elsewhere in low- and middle-income countries [10, 15, 34]. Integrated care enhances equity and access to care as noted in Malawi, South Africa, Swaziland and Kenya [35] as it enables patients to receive services under the same roof as a ‘*one stop shopping Centre’* [36]. Generally, integration offered more clinical benefits in resource-limited countries where NCD services are not yet routinely available.

Integration in our study provided unique opportunities to foster early management and prevention of disease progression. These findings are in line with what was reported elsewhere in Tanzania, Kenya, Malawi, and Nigeria [18, 37–39]. However, some challenges have been associated in integrated care model, including reduced knowledge and utilisation of the specific services, increased waiting time due to staff shortages and problems relating to patient flow through the system [40–43]. Also, even though there are potential strong benefits associated with integration of services, this system re-organisation could jeopardise HIV programmes.

Stigma reduction is an important aspect of acceptability. Whereas HIV and most of clients with co-morbidities viewed integrated care as a stigma reduction strategy, a minority of NCD clients either with HT of DM were unhappy with the model mainly because of structural challenges at the health facilities, which were too open and consultation rooms were few and small, which undermined confidentiality and privacy. Greater efforts are, therefore, needed to address privacy and confidentiality issues that were reported as barriers at the lower level and periphery health facilities. Although there were notable improvements in medicine availability, the frequent stockouts observed by a few NCD patients, the supply chain and their associated costs are priority areas warrant greater health system attention and health policy considerations [44].

### Study strengths and limitations

This is the first study of its kind in Tanzania that informs about the acceptability of an integrated service delivery model. Our study has several strengths. First, our study took a three phases pre-, mid-, and end-line phases which are consistent with the TFA, hence making it possible to document and assess how perceptions evolved over time as the integration of services was operationalised. We recruited patients with different disease conditions as the end-users of the health system services to judge the acceptability from varied disease and co-morbid specific angles. Thirdly, we triangulated the data across perspectives from patients and health care providers interviews, and observations. However, our study size was small hence requiring replication on a larger scale.

## Conclusion

This study illustrates strong levels of acceptability of the integrated care delivery model to both patients and health care providers. The structure, the pathway, the friendly environment, patient-provider relationship, time and cost saving, and provision of joint education have all attributed to such a level of acceptability. Challenges, however, included long waiting times and limited privacy in lower and periphery health facilities due to infrastructural challenges. For sustainability and scalability concerted efforts are warranted to ensure maximum privacy and confidentiality for all patients receiving the service in all levels of health facilities where integrated care model is implemented. However, replication in a larger comparative study is needed to confirm these findings before recommending for a wider scale.

## Data Availability

The data set used for the analysis in this manuscript is available from the corresponding author on reasonable request.

## Abbreviations

CTC: Care and Treatment Centre
DM: Diabetes Mellitus
HT: Hypertension
IDIs: In-depth Interviews
MOCCA: Models of Chronic Care in Africa
NCD: Non-Communicable Diseases
OPD: Out-Patients Departments
PLHIV: People Living with HIV
TFA: Theoretical Framework of Acceptability
